# Arthroscopic Fixation of a Posterior Acetabular Wall Fracture: A Case Report

**DOI:** 10.7759/cureus.6264

**Published:** 2019-11-30

**Authors:** Tahsin Gürpınar, Barış Polat, Enes Kanay, Ayşe Polat, Yusuf Öztürkmen

**Affiliations:** 1 Orthopaedics and Traumatology, Istanbul Training and Research Hospital, Istanbul, TUR; 2 Orthopaedics and Traumatology, University of Kyrenia, Kyrenia, CYP; 3 Orthopaedics and Traumatology, Beykoz State Hospital, Istanbul, TUR; 4 Orthopaedics and Traumatology, Dr. Akçiçek State Hospital, Kyrenia, CYP

**Keywords:** acetabular posterior wall fracture, arthroscopic reduction, hip arthroscopy

## Abstract

The aim of this study was to present the results of an unusual surgical technique for the treatment of posterior wall acetabular fractures. A 49-year-old man presented to the emergency department after a fall from three meters. His X-rays revealed a right acetabular posterior wall fracture. He was treated with arthroscopic reduction and fixation using a cannulated screw through arthroscopic portals. The patient was allowed partial weight-bearing immediately and had a satisfactory outcome. In selected cases, arthroscopic reduction and fixation in acetabular posterior wall fractures could be a good surgical option with the advantages of simultaneous labral treatments and loose body removal.

## Introduction

The treatment of intra-articular acetabular fractures is challenging and furthermore, traditional open approaches require wide dissection with considerable morbidity and complication rates. Even conservative treatments require traction of the extremity and/or prolonged bed rest which is prone to decubitus ulcers and an increased risk of thromboembolism. In order to avoid these complications and invasive surgery, minimally invasive techniques have been tried such as percutaneous fixation with the assistance of CT and fluoroscopy [1].

The indications of hip arthroscopy have been evolving, and it appears to be effective and safe in the setting of trauma [2]. Arthroscopy has been used to check the reduction of the acetabular fracture while percutaneously inserting screws [3]. In addition, it has been used to extract posttraumatic loose fragments. However, to the author’s knowledge, there have been only two reports where the acetabular fracture is only fixed arthroscopically through arthroscopic portals [4,5]. The authors stated that arthroscopy can be a reasonable option in selected cases. This study reports a case of an acetabular posterior wall fracture, which was reduced and fixated through arthroscopic portals.

## Case presentation

A 49-year-old man presented to the emergency department after a fall from three meters. In his initial assessment, he was hemodynamically stable and no visceral or cerebral injuries were diagnosed. X-rays revealed an L2 vertebral fracture, a right scapula fracture, and a right acetabular posterior wall fracture. Since the scapular fracture was non-displaced, conservative treatment was considered. The L2 vertebral fracture was unstable, and thus surgical treatment was planned. On the other hand, the posterior acetabular wall fracture was minimally displaced and, based on the patient’s multiple trauma and the morbidities of prolonged bed rest, arthroscopic fixation of the posterior wall was planned. The patient was informed, and consent was obtained for the surgery and scientific purposes.

**Figure 1:**
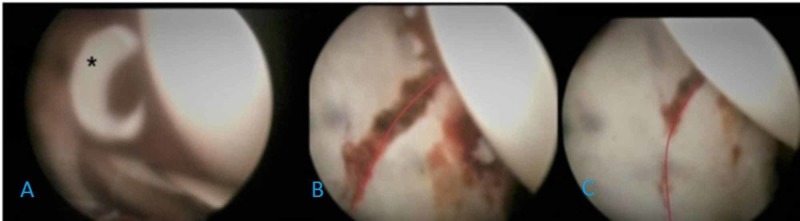
Arthroscopic images of surgery. A: * Intra-articular chondral fragments debrided from the joint. B: The red line shows the fracture line before reduction. C: The red line shows the fracture line after reduction.

**Figure 2:**
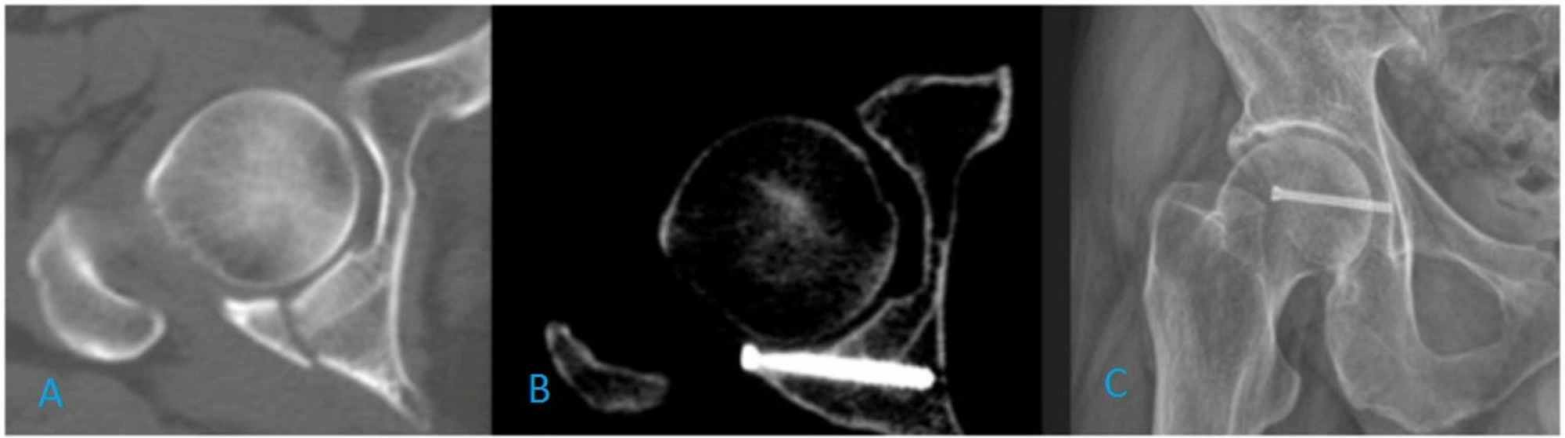
A: Preoperative axial CT scan image of the hip. B: Postoperative first year axial CT scan of the hip. C: Postoperative first day X-ray of the hip.

Under general anesthesia, posterior short-segment instrumentation and fusion for the L2 vertebral fracture were performed and the patient was subsequently taken to the fracture table in the supine position. Traction was applied to distract the hip joint and to reduce the fracture fragment properly. After the establishment of the standard anterolateral (AL) portal, the anterior and the posterolateral (PL) portals were established under direct visualization. Hemarthrosis was drained and the joint was irrigated. After obtaining a clear view, small chondral free particles and labral small tears were observed and they were debrided from the joint (Figure 1A). The capsulotomy was only performed between the AL and PL portals to avoid extravasation and instability. Care was also taken to maintain low intra-articular pressure (30-35 mm Hg) in order to prevent fluid leakage. The posterior labrum was left intact but detached from the capsule to reach the extra-articular surface of the posterior wall. Since the fracture was at a posterior wall, the main working portal was PL portal and the view is obtained mostly through the AL portal. The fracture line was visualized, and reduction of the posterior wall was achieved by traction and manipulation of K wire from the PL portal (Figure 1B, 1C). Under direct visualization, the fracture was fixed with a K wire and an obturator oblique view confirmed the position of the wire. Final fixation was performed with a 4.5 mm cannulated screw through the PL portal, and the fracture line was compressed. Following the complete diagnosis of the joint, traction was released and the portals were closed. The postoperative X-ray and CT confirmed the reduction (Figure 2A-2C). After the surgery, a first-generation cephalosporin was administered, partial weight-bearing was permitted, and no complications were seen. Since it was a small posterior wall fracture that was relatively stable and body mass index of the patient was low, we started weight-bearing as tolerated in a short time and the patient was able to weight bear in four weeks. This rehabilitation is not our standard protocol but individualized for this patient. At the final follow-up one year after surgery, there has been no displacement on X-ray or CT. The patient had a full function without any pain, and the Harris hip score was 100. 

 

## Discussion

Arthroscopic techniques have been successfully used for the treatment of intra-articular fractures of different joints for a long time. They have the advantages of direct and magnified visualization of the intra-articular space and decreased invasiveness. Additionally, soft tissue and cartilage problems can be addressed simultaneously [6]. However, arthroscopic treatment for traumatic acetabular fractures is relatively uncommon. In this particular case, surgery was only performed through arthroscopic portals.

The treatment of posterior wall fractures of the acetabulum can be a challenging issue. Non-operative treatment generally consists of bed rest and traction. This prolonged bed rest can lead to bedsores, pin track infections, thromboembolism, pneumonia, and urinary infections. On the other hand, open reduction of these fractures requires wide dissection, which is prone to prolonged recovery and heterotrophic ossification (HO). Besides, HO generally appears in patients requiring surgical dissection of gluteal muscles [7]. At this point, arthroscopic surgery has the advantage of early weight-bearing and range of motion, while avoiding wide dissection. Screw penetration of the hip joint following acetabular fracture fixation is a relatively uncommon and complicated procedure; it may have a catastrophic effect on the postoperative function of the hip joint. In addition, to prevent articular surface screw penetration, the quality of reduction can be directly visualized by the arthroscope.

Several studies have reported that traumatic injuries of the hip frequently result in considerable intra-articular pathologic changes, including loose bodies, labral tears, and osteochondral lesions [6]. Similarly, chondral free particles and labral damage were identified in this case. Osteoarthritis following hip dislocation and fracture dislocations was caused by the intra-articular presence of free osteochondral fragments. Additionally, Evans et al. proved experimentally that free cartilaginous particles inside the joint increase chondrolytic enzyme activity and induce secondary arthrosis [8]. As a result, the free particles were removed and the labrum was divided since they could cause the progression of traumatic osteoarthritis. Besides, many studies have reported that radiographs and even CT scans can underestimate the true incidence of pathologic intra-articular findings [9]. Therefore, arthroscopy is not only helpful for treating these concomitant problems but also is beneficial for identification.

This procedure is, however, not free of complications. In acetabular fractures, the concerns are particularly related to fluid extravasation, which could lead to life-threatening complications [10]. Bartlett et al. reported an intra-abdominal compartment syndrome that presented as cardiopulmonary arrest and therefore, they did not advocate the arthroscopic procedures for acute or healing acetabular fractures [10]. On the other hand, in a recent review, hip arthroscopy in trauma was considered as a safe and effective treatment option [2]. In this case, low intra-articular pressure was delicately maintained and no symptoms of abdominal fluid extravasation were observed. In the clinic, abdominal ultrasonography is routinely performed after all hip arthroscopy cases,in and the amount of intra-abdominal fluid, in this case, was not substantially different from the regular cases.

## Conclusions

In selected cases, arthroscopic reduction and fixation in acetabular posterior wall fractures rather than the conservative or open surgical treatment could be a good surgical option with the advantages of simultaneous labral treatments and loose body removal.
